# Risk of HIV viral rebound in HIV infected patients on direct acting antivirals (DAAs) treatment for HCV

**DOI:** 10.1371/journal.pone.0262917

**Published:** 2022-02-03

**Authors:** Giulia Morsica, Laura Galli, Emanuela Messina, Antonella Castagna, Sabrina Bagaglio, Stefania Salpietro, Della Torre Liviana, Caterina Uberti-Foppa, Hamid Hasson

**Affiliations:** 1 Division of Infectious Diseases, IRCCS San Raffaele Scientific Institute, Milan, Italy; 2 Vita-Salute University, Milan, Italy; National Taiwan University Hospital, TAIWAN

## Abstract

**Background:**

The dynamic of HIV-viral load (VL) remains poorly investigated in HIV/HCV patients under direct acting antivirals (DAAs).

**Methods:**

We retrospectively evaluated HIV-VL at baseline (BL) during and up to 24 weeks post-DAAs in a cohort of 305 HIV-1/HCV patients, on ART and with no HIV virological failure (VF) in the 6 months before treatment with DAAs; during the period of observation VF was defined as confirmed VL≥50 copies/mL; virological blips (VB, transient, not confirmed, VL ≥50 copies/mL). Stepwise Cox regression models were fitted to estimate adjusted hazard ratios (aHR) of VF.

**Results:**

Fifteen VF occurred in 13 patients over 187 person-years of follow-up (PYFU): incidence rate (IR) of 8.0 per 100-PYFU (95% CI = 4.0–12.1); 29 VBs were detected in 26 patients over 184 PYFU: IR = 15.8 per 100-PYFU (95% CI = 10.0–21.5). The most prominent factor associated with VF was the presence of BL HIV residual viremia (RV = HIV-RNA detectable but not precisely quantifiable) [aHR = 12.26 (95% CI = 3.74–40.17), P<0.0001]. Other factors were ≥1 VBs in the 6 months before DAAs [aHR = 6.95 (95% CI = 1.77–27.37) P = 0.006] number of ART regimens failed before DAAs initiation [aHR (per more regimen) = 1.22 (95% CI = 1.04–1.42), P = 0.012] and age [aHR (per year older) = 1.16 (95% CI = 1.04–1.29), P = 0.010].

**Conclusions:**

Our findings underline the importance for close monitoring HIV-VL in selected patients. Whether this phenomenon is triggered by the rapid clearance of HCV remains to be established.

## Introduction

There are approximately 2,200,000 individuals worldwide living with chronic hepatitis C (HCV) and human immunodeficiency virus (HIV) co-infection [1]. Patients infected with HIV and HCV have higher HCV viral loads, decreased rates of HCV clearance after acute infection, and higher rates of decompensated cirrhosis and hepatocellular carcinoma development compared to the counterpart of HCV monoinfected patients [2–4].

From an immunopathological point of view, HCV has shown to negatively affect HIV infection by leading to an increase in soluble markers of systemic inflammation and the production of inflammatory cytokines and chemokines [5–7]. In regard of viral interaction between these two viruses, Baroncelli et al. [8] showed that HCV infection favours the persistence of low-level HIV viremia between 18 and 24 months from the start of the raltegravir-based regimen. The study by Pugliese et al. [9] showed that replicative HCV infection predicted virological failure in HIV patients on stable ART. Altogether, these data suggest a complex interplay between HIV and HCV infection and the need for HCV treatment intervention. In recent years, the outcomes of HIV–HCV co-infected patients have improved with the introduction of direct acting antivirals (DAAs), with more than 90% of treated subjects cured considering clinical trials as well as real world longitudinal studies [10–16]. While these studies focused on the efficacy and safety of new direct acting antivirals (DAAs) for HCV treatment, few data (the majority of whom coming from clinical trials) are available on the dynamic of HIV viremia during and after DAAs treatment [17–20].

Due to the scarce literature on the topic of virological control of HIV infection during modern anti-HCV treatment, we decided to focus our investigation on the frequency of HIV rebound and possible associated factors, by performing a longitudinal evaluation of HIV-viremia during and after DAAs treatment.

## Methods

This is an observational, retrospective, cohort study, performed in HIV/HCV positive patients, attending the Infectious Diseases Department of San Raffaele Scientific Institute, treated with DAAs during the years 2015–2018 with no previous DAAs exposure, on HIV treatment for at least 1 year before DAAs and no HIV virological failure (VF) in the 6 months before DAAs treatment.

This study was approved by the ethics committee of the San Raffaele Scientific Institute, Milan, Italy. At their first visit in our center, patients signed an informed consent (also approved by the ethics committee of the San Raffaele Scientific Institute) to use their data for research purposes and to be included in the database of our department (CSLHIV-Cohort). Recorded data are anonymised and managed according to the Good Clinical Practice Guidelines published by the World Medical Association Declaration of Helsinki.

Epidemiological, clinical and laboratory parameters at baseline (BL corresponding to date of initiation of DAAs) up to W24-post DAAs treatment were used in the analyses.

Plasma HIV-1 RNA concentrations were measured with a real-time PCR system (Abbott Molecular, Des Plaines, IL, USA). The real-time PCR assay has three possible outputs: (i) a quantitative result for HIV-1 RNA values ≥40 copies/mL; (ii) a semi- quantitative result (detectable <40 copies/mL) when HIV-1 RNA is detectable but not precisely quantifiable; and (iii) a qualitative result (‘undetectable’) when no signal can be detected. HIV-1 RNA was thus defined as undetectable when no signal could be detected (“target not detected”); residual viremia (RV) was defined as any detectable PCR signal *<*50 HIV-1 RNA copies/mL as assessed by Abbott real-time PCR [21].

Virological failure was assessed by the detection of 2 consecutive HIV-1 RNA determinations ≥50 copies/mL after BL, in the period from week (W) 4 to W24 post-DAAs treatment. Virological blips (VB) were defined by the occurrence of a single HIV-1 RNA value >50 copies/mL after BL, in the same time-window of VF definition (from W4 to W24 post-DAAs treatment). Viral suppression (VS) was defined as HIV-RNA <50 copies/mL from baseline evaluation (DAAs initiation) to end of the follow-up (24 weeks post-DAAs treatment). Within the definition of viral suppression were identified those patients with residual viremia (HIV-RNA <50 copies ml, but with detectable HIV-load between 1–49 copies).

Hepatitis C virus-RNA was quantified by a real-time PCR system (Abbott Molecular, Des Plaines, IL, USA): the lower limit of quantification was 12 IU/mL.

HCV genotyping was performed by routine laboratory assay involving direct sequencing of the non-structural region (NS) 5b followed by phylogenetic analysis. HIV resistance was determined by commercially available methods and as part of routine laboratory tests.

Drug resistance mutations were identified using the Genotypic Resistance Interpretation Algorithm of the Stanford HIV Drug Resistance database Program (version 8.9–1, last updated on 2019-10-25; http://hivdb.stanford.edu).

Liver fibrosis degree was assessed by stiffness measurement in kilopascal (kPa) using transient elastography (Fibro Scan®, Echosens, Paris, France) and according to metavir score. We classified liver fibrosis using a stiffness limit of 9.4 kPa for fibrosis stage from F0 to F2, 9.5 kPa for fibrosis stage 3, and 12.5 kPa for fibrosis stage 4.

Patients’ characteristics were described as median (interquartile range, IQR) for continuous variables or proportions for categorical variables. Continuous variables were compared using the Kruskal-Wallis test or the Wilcoxon rank sum test, as appropriate. Differences between proportions were tested by the chi-square or Fisher’s exact test.

The incidence rates of VF and VB were calculated by use of univariable Poisson regression models; rates were reported as the number of VF (primary endpoint) and VB per 100 person-years of follow-up (PYFU) with the corresponding 95% confidence interval (95% CI).

Time to VF was censored at the date of first VF, lost to follow-up, death or data freezing (September 12, 2019), whichever occurred first.

Multivariable analysis was performed by use of the Cox regression model to assess factors associated with the risk of VF. Variables known to have a potential effect on this outcome or those with p<0.1 in exploratory analysis were considered to obtain the final multivariable model and included: age, gender, HIV risk factor, nadir CD4+ T cells count, HBV co-infection, years of ART, type of BL ART regimen, HCV genotype, interferon naïve, previous HIV virological failure before DAA, previous HIV virological blip in the 6 months before DAA, liver stiffness, HCV-RNA levels, aspartate amino transferase (AST), alanine amino transferase (ALT) levels, HIV-1 RNA load, CD4 T cells count. A stepwise variable selection algorithm with entry and stay criteria of 0.10 and 0.05, respectively, was applied; adjusted hazard ratios (aHR) of VF were reported with the corresponding 95% CI for significant covariates. We assessed the proportional hazards assumption for the Cox model by adding time-dependent variables to the original final model (i.e. the product of each factor significantly associated with VF and logarithm of time); as no statistically significant p-values were observed, the assumption of proportional hazards was satisfied for all the considered covariates.

All analyses were conducted using SAS statistical software version 9.4 (Statistical Analyses System Inc, Cary, NC, USA).

## Results

Of 511 HIV-1 infected patients treated with DAAs at the San Raffaele Hospital during the years 2015–2018, 305 met the inclusion criteria and were included in the present study.

The median number of HIV-1 RNA determinations per patient was 5 (IQR 5–6).

Fifteen VF occurred in 13 patients during 187 person-years-follow-up (PYFU) for an estimated incidence rate (IR) of 8.0 per 100-PYFU (95% CI 4.0–12.1); 26 patients had 29 VBs during 184-PYFU, for an estimated IR of 15.8 per 100-PYFU (95% CI 10.0–21.5). Main clinical characteristics of HIV/HCV co-infected patients at baseline (BL) according to virological trend during follow-up (VF or VB or maintenance of VS) are summarized in Table 1.

**Table 1:** Baseline characteristics of the 305 HIV/HCV infected patients according to HIV virological outcome during or after DAAs for HCV infection.

Characteristics	Category	VB^§^	VF^§^	VS	P-value^§§^
		(N 25)	(N 13)	(N 267)	
**Demographic data**					
Age, years		53.0 (49.7–56.3)	55.5 (53.9–57.7)	52.9 (50.3–55.4)	0.097
Male gender		22 (88.0%)	8 (61.5%)	193 (72.3%)	0.150
**HIV related data**					
Risk factor for HIV-1 infection	Heterosexual	1 (4.0%)	1 (7.7%)	17 (6.4%)	0.256
	Homosexual	5 (20.0%)	0 (0%)	53 (19.8%)	
	EX-TD/TD	18 (72.0%)	8 (61.5%)	152 (57.0%)	
	Other/Unknown	1 (4.0%)	4 (30.8%)	45 (16.8%)	
Nadir CD4+ T cells count, number/mmc		211 (130–296)	131 (50–276)	197 (103–301)	0.354
Years of HIV-1 infection		26.3 (18.5–30.0)	29.7 (19.8–30.9)	27.0 (17.5–30.9)	0.545
ART duration, years		16.3 (10.9–21.5)	20.6 (17.0–23.9)	19.3 (13.4–23.2)	0.091
Number of failed ART regimens before DAA		1 (1–3)	5 (3–8)	2 (0–5)	0.019
Type of ART at DAA start	Monotherapy	2 (8.0%)	0 (0%)	13 (4.8%)	0.306
	Dual therapy	5 (20.0%)	2 (15.4%)	62 (23.2%)	
	2 NRTIs + 1 PI	6 (24.0%)	4 (30.8%)	29 (10.9%)	
	2 NRTIs + 1 NNRTI	1 (4.0%)	0 (0%)	29 (10.9%)	
	2 NRTIs + 1 INSTI	8 (32.0%)	5 (38.5%)	110 (41.2%)	
	Other ART regimens	3 (12.0%)	2 (15.4%)	24 (9.0%)	
ART change pre-DAA	Yes	7 (28.0%)	3 (23.1%)	108 (40.4%)	0.236
	No	18 (72.0%)	10 (76.9%)	159 (59.6%)	
CD4+ T cells count, number/mmc		554 (316–817)	700 (439–1033)	666 (477–838)	0.288
CD8+ T cells count, number/mmc		837 (699–988)	1136 (858–1456)	751 (568–1029)	0.019
CD4+/CD8+ ratio		0.57 (0.39–0.87)	0.49 (0.38–1.07)	0.83 (0.59–1.23)	0.007
HIV-1 RNA	Undetectable	18 (72.0%)	5 (38.5%)	240 (89.9%)	<0.0001
	Residual viremia	7 (28.0%)	8 (61.5%)	27 (10.1%)	
HIV-1 RNA blip in the 6 months before DAAs	No	24 (96.0%)	10 (76.9%)	259 (97.0%)	0.001
	Yes	1 (4.0%)	3 (23.1%)	8 (3.0%)	
**HBV/HCV related data**					
HbsAg	Positive	1 (4.0%)	1 (7.7%)	10 (3.7%)	0.691
	Negative	22 (88.0%)	10 (76.9)	240 (89.9%)	
	Unknown	2 (8.0%)	2 (15.4%)	17 (6.4%)	
HCV-RNA, Log_10_ IU/mL		5.91 (5.2–6.23)	5.97 (5.21–6.22)	5.94 (5.47–6.32)	0.461
ALT Levels, IU/L		59 (44–102)	81 (70–102)	69 (43–119)	0.602
AST Levels, IU/L		50 (32–81)	64 (40–87)	54 (36–91)	0.684
Calendar Year DAAs		2016 (2015–2018)	2016 (2015–2017)	2017 (2015–2018)	0.152
DAA duration, weeks		12 (12–24)	24 (12–24)	12 (12–16)	0.023
Type of DAAs	VEL+SOF	10 (40.0%)	2 (15.4%)	97 (36.3%)	0.070
	DAC+SOF	6 (24.0%)	4 (30.8%)	25 (9.3%)	
	LED+SOF	3 (12.0%)	4 (30.8%)	36 (13.4%)	
	SOF+RBV	2 (8.0%)	2 (15.4%)	10 (3.7%)	
	GLE+PIB	2 (8.0%)	0	33 (12.4%)	
	ELB+GRA	0	1 (7.7%)	20 (7.5%)	
	SIM+SOF	1 (4.0%)	0	32 (11.9%)	
	EXV+OMB+PAR+RTV	1 (4.0%)	0	11 (3.7%)	
	OMB+PAR+RTV	0	0	3 (1.1%)	
Use of RBV in the DAA regimen	No	10 (40.0%)	4 (30.8%)	139 (52.1%)	0.185
	Yes	15 (60.0%)	9 (69.2%)	128 (47.9%)	
HCV genotype	1a	9 (36.0%)	3 (23.1%)	107 (40.1%)	0.800
	1b	0 (0%)	0 (0%)	25 (9.4%)	
	1c	1 (4.0%)	2 (15.4%)	15 (5.6%)	
	2a	0 (0%)	1 (7.7%)	6 (2.2%)	
	2b	0 (0%)	0 (0%)	1 (0.4%)	
	2c	1 (4.0%)	0 (0%)	3 (1.1%)	
	3	0 (0%)	0 (0%)	1 (0.4%)	
	3a	9 (36.0%)	4 (30.8%)	58 (21.7%)	
	4	0 (0%)	0 (0%)	6 (2.2%)	
	4a	2 (8.0%)	1 (7.7%)	15 (5.6%)	
	4c	0 (0%)	0 (0%)	1 (0.4%)	
	4c,4d*	2 (8.0%)	0 (0%)	8 (3.0%)	
	4d	1 (4.0%)	2 (15.4%)	21 (7.9%)	
Liver stiffness, kPa		13.3 (7.4–21.3)	12.2 (11.7–14.6)	8.8 (5.5–17.3)	0.043
Fibrosis degree according to metavir score					0.003
	F0-F2	9 (36.0%)	1 (7.7%)	140 (52.4%)	
	F3-F4	16 (64.0%)	12 (92.3%)	127 (47.6%)	
Cirrhosis		15 (60.0%)	6 (46.0%)	94 (36.0%)	0.041

Abbreviations: ART = antiretroviral therapy; NRTIs = nucleoside reverse transcriptase inhibitors; PI = protease inhibitors;

INSTI = integrase strand transfer inhibitors; VB = HIV viral blip; VF = HIV virological failure; VS = viral suppression.

Results described as median (quartiles) or frequency (%).

^§^ One subject with both VF and VB during follow-up is included in the VF group.

^§§^ by Kruskal-Wallis test (continuous variables) or chi-square test (categorical variables).

* On the basis of phylogenetic analysis, in these cases it was not possible to distinguish between GT 4c and 4d.

With regard to demographic and HIV related data, by univariate analysis, no differences emerged concerning the distribution of age, sex and risk factor for HIV-1 infection among VB, VF, VS. No difference in the type of BL ART regimens or ART duration was observed among the three groups of patients.

In the 6 months before BL evaluation, ART was modified in order to avoid drug-drug interactions in 7 (27%) VB, in 3 (25%) VF and 108 (40%) VS patients (P = 0.236).

Patients with VF had a higher number of ART-failure before DAAs (median = 5, IQR 3–8) compared to VB patients (median = 1, IQR 1–3) and VS patients (median = 2, IQR 0–5) (P = 0.019).

One/25 (4%) VB patients, 3/13 (23%) VF and 8/267 (3%) VS patients had at least 1 VB in the 6 months before DAAs treatment, this finding reaching statistical significance (P = 0.001).

Concerning the baseline immunological status (Table 1), the CD4 nadir T cells count (P = 0.354) and CD4 T cells count (P = 0.288) were not different among VB, VF and VS patients, while CD4/CD8 ratio (P = 0.007) and CD8 T cells count were found to be significantly different among the three groups [median value 1136 (IQR 858–1456) in VF vs. 837 (IQR 699–988) in VB and 751 (IQR 568–1029) in VS; P = 0.019].

Residual viremia (detectable PCR signal *<*50 HIV-1 RNA copies/mL) at BL was more frequently present among patients who had VF respect to VB and VS patients (61.5% vs. 28.0% and 10.1%, respectively; P<0.0001).

With regard to HBV/HCV related data, HBsAg, HCV-RNA load and necroinflammatory activity (assessed by transaminases value), were similarly distributed among the different groups of patients classified on the basis of HIV virological trend (see Table 1).

During a median follow-up since DAA start to last visit of 8.2 (5.6–8.3) months, the median number of HIV-1 RNA determinations per patient was 5 (IQR 5–6).

Patients with VF were treated for a longer period with DAAs as compared to those with VB or VS (overall comparison P = 0.023; VF vs. VS = 0.016. and had a higher number of HIV viremia determinations: 8 (IQR 5–8) in VF, vs. 6 (IQR 5–7) in VB, and 5 (IQR 5–6) in VS, (overall comparison: P<0.0001; VF vs. VS, P = 0.0007; VB vs. VS P = 0.0004). Detectable HIV load (≥50 copies/mL) was revealed in 7/13 (54%) VF patients within 12 weeks from DAAs initiation.

No difference in type of DAA (P = 0.070) or use of RBV (P = 0.185) was found in these three groups of patients (VF vs. VB vs. VS).

A higher stiffness value was detected in VB and VF patients respect to VS patients (P = 0.043); with regard to fibrosis degree, patients with VF and VB showed more frequently advanced liver disease (F3 or F4 fibrosis degree according to metavir score) than VS patients (P = 0.003). In total 115/305 (37.7%) patients had cirrhosis by transient elastography examination [60% (n = 15) vs. 46% (n = 6) and 36% (n = 94) in VB, VF and VS, respectively; P = 0.041].

We also evaluated the clinical characteristics of patients at the end of treatment with DAAs (ETR-DAAs).

At ETR-DAAs, 32% subjects with VB profile and 38.5% patients with VF had HIV-RNA >50 copies/mL; at the same time point, RV was detected in 28% of VB, 23% of VF and 13.60% of VS, while HIV viremia was undetectable (no signal detection) in 40% of VB, 38.5% of VF and 86.4% of VS patients (P< 0.0001); CD4/CD8 ratio [median value 0.6 (IQR 0.45–0.97) vs. 0.52 (IQR 0.43–0.80) and 0.84 (0.6–1.17); P = 0.011] but the absolute number in CD4 T cells count and CD8 T cells count were found not to be lower in VB and VF patients respect to VS patients.

With regard to anti-HCV treatment outcome, all VF and VB patients were cured for HCV infection, while 265/267 (99%) VS patients had a sustained virological response.

We subsequently evaluated a number of factors possibly associated with VF and we identified, by multivariable analysis, the presence of HIV residual viremia [aHR = 12.26 (95% CI = 3.74–40.17), P<0.0001] at baseline, as the main factor associated with VF. Other variables associated with VF were the presence of ≥1 VB in the 6 months before DAAs start [aHR = 6.95 (95% CI = 1.77–27.37) P = 0.006], number of ART regimens failed before BL [aHR (per more regimen) = 1.22 (95% CI = 1.04–1.42), P = 0.012] and age [aHR (per year older) = 1.16 (95% CI = 1.04–1.29) P = 0.010].

Seven/13 patients (PT1, PT3, PT 5, PT6, PT11, PT12, PT13) with VF had a resistance test at failure and drug-induced resistance was observed in 5 of these 7 patients all infected by HIV subtype B: three patients (PT6, PT12 and PT13) subsequently changed ART treatment according to resistance pattern; one patient (PT3) had resistance associated mutations (RAMS) with high level resistance to ABC and 3TC, but no resistance to protease inhibitors, and the physician decided to wait before changing treatment. Interestingly, the patient (PT1) with high level resistance to raltegravir re-suppressed up to the last visit in 2019, without drug switch. The remaining two patients (PT5 and PT11) had a wild-type virus and did not modify their ART regimen.

In 2 (PT7 and PT10) of the 6 patients who did not perform a resistance test, poor adherence to ART during DAAs treatment was reported; therefore, were re-counselled on the importance to adhere to their ART prescription.

Of the remaining 4 patients without a resistance test, PT2 and PT9 changed ART, PT4 and PT8 maintained their ART regimen. Virological data for these 13 patients are summarized in Table 2.

**Table 2:** Drug regimens and resistance profile in patients with HIV VF during or after DAAs treatment (within 24 weeks post treatment).

Patient ID	DAAs duration, weeks	HIV load at VF, copies/mL	Week of VF	ART regimen at VF	Type of resistance test	Resistance mutations	Resistance degree to current ART^§^	ART change
**1**	**24**	**152**	**12**	**RAL, TAF, FTC**	**INSTI**	**N155HN, T97AT, D232DN**	**high level resistance**	**no**
							**RAL**	
**2**	**24**	**517**	**4**	**LPV/r, TDF, FTC**	**-**	**-**	**-**	**yes**
**3**	**24**	**177**	**12**	**DRV/r, ABC, 3TC**	**RTI, PI**	**L10I, M41L, D67N, K70R, M184V, T215Y**	**high level resistance**	**no**
							**ABC**	
							**3TC**	
**4**	**12**	**121**	**16**	**DTG, ABC, 3TC**	**-**	**-**	**-**	**no**
**5**	**24**	**339**	**29**	**DTG, ABC, 3TC**	**INSTI**	**none**	**-**	**no**
**6**	**24**	**97**	**12**	**RAL, DRV/r, TDF, FTC,**	**INSTI**	**N155H, E92EQ**	**high level resistance**	**yes**
							**RAL**	
**7** *	**12**	**110784**	**8**	**RAL, TAF, FTC**	**-**	**-**	**-**	**no**
**8**	**12**	**174**	**13**	**EVG/c, FTC, TDF**	**-**	**-**	**-**	**no**
**9**	**24**	**95**	**24**	**DTG, ETR**	**-**	**-**	**-**	**yes**
**10** *	**12**	**1835**	**24**	**RAL, ABC, 3TC**	**-**	**-**	**-**	**no**
**11** ^#^	**12**	**146**	**12**	**ATV/r, TAF, FTC**	**RTI, PI, INSTI**	**none**	**-**	**no**
**12**	**24**	**95**	**29**	**LPV/r, TDF, FTC**	**RTI, PI**	**M46L, I84V, L90M, L10I, A71V, M41L,D67N,T69D, L210W,T215Y,K219R, A98G,K103N,Y181C,G190A,H221Y**	**high level resistance**	**yes**
							**LPV/r**	
							**TDF**	
							**low level resistance**	
							**FTC**	
**13**	**12**	**175**	**4**	**DRV/r, TDF, FTC**	**RTI, PI**	**M184V**	**high level resistance**	**yes**
							**FTC**	

Abbreviations: DAAs = direct acting antivirals; ART = antiretroviral therapy; RAL = raltegravir; TAF = tenofovir alafenamide; FTC = emtricitabine; LPV = lopinavir; r = ritonavir; c = cobicistat; TDF = tenofovir disoproxil fumarate; DRV = darunavir; ABC = abacavir; DTG = dorultegravir; 3TC = lamivudine; EVG = eviltegravir; ETR = etravirine; RTI = reverse transcriptase inhibitors, PI = protease inhibitors; INSTI = integrase strand transfer inhibitors.

^§^ Levels of drug resistance were inferred using the Genotypic Resistance Interpretation Algorithm of the Stanford HIV Drug Resistance database Program (version 8.9–1, last updated on 2019-10-25; http://hivdb.stanford.edu).

*Patient 7 and 10 were poorly adherent to ART; PT 10 did not perform regular monitoring of HIV-RNA during DAAs treatment.

^#^Resistance test was performed on DNA n PT11.

Concerning patients with VB, only one of 28 patients performed a resistance test showing no RAMs. All these patients maintained viral suppression in the period of observation (up to 24 W post DAAs treatment) and did not switch their ART regimen.

## Discussion

In this analysis, performed in a cohort of HIV infected patients in whom we investigated the frequency and the risk factors associated with HIV viral rebound during and after DAAs, we found that HIV-VF was not a so rare event, occurring in 8.0 per 100 person years of follow-up and was mainly related to the dynamic of virological control, assessed by RV at BL, presence of virological blips before DAAs treatment and number of previous failed ART regimens.

By univariate analysis we found that patients with VF had longer DAAs duration with a higher number of HIV viremia determination. However, 54% VF was revealed within 12 weeks from initiation of DAAs treatment.

A longer duration of DAAs in VF was the consequence of a higher fibrosis degree assessed by metavir score (F3-F4) respect to those with VB and VS. Liver fibrosis has been observed in the absence of HBV, HCV, ART in HIV monoinfected patients with poor control of HIV viremia [22, 23]. Additionally, several studies showed that the control of HIV replication by ART prevents the progression of liver damage [24, 25].

In this regard, in our group of HIV/HCV coinfected patients with HIV VF during DAAs treatment, we found a higher frequency of previous ART failure respect to patients with VB and those with persistent VS, that could have contributed to liver damage.

A higher baseline CD8 T cells count was found in VF patients respect to VB and VS patients. Although we have not a clear explanation for this phenomenon, we hypothesized that CD8 cells elevation in VF was consequent to viral reservoir mobilization or activation of replicative-competent virus.

By multivariate analysis we found that the estimated rate of VF was higher than that reported by Rusconi et al. [26] on HIV-1 infected patients on combination-ART, indicating an overall incidence rate of VF (with a threshold of 50 HIV-RNA copies for the definition of VF) of 2.08 PYFU (95% CI: 1.93–2.22) during a mean follow-up of 4.1 years.

One other study [27] reported 3.48 virological failure per 100 PYFU (95% CI 3.33–3.64) including patients with persistent viral suppression (HIV-1 load <50 copies/mL), patients experiencing low level viremia (LLV) 50–199 copies/mL and patients experiencing LLV 200–499 copies/mL, during a median follow-up of 2.3 years.

In another study, Teira et al., [28] reported an incidence rate of VF corresponding to 2.34 cases per 100 PYFU in fully suppressed patients, during a median period of 1.7 years, using a threshold of 200 copies/mL for the definition of HIV-VF.

All these studies were performed in larger samples than the one evaluated in the present study. However, a characteristic that differentiates our study from those previously cited, is that we included exclusively HIV/HCV infected patients being also under DAAs treatment. In the report by Rusconi et al. [26], the prevalence of HCV co-infection was 14%, while this data was not reported in the other two studies [27, 28].

Therefore, the different characteristics of patients as well as the difference in the threshold for the definition of VF in two [27, 28] of these prior studies, may be responsible for the discrepancy in results.

There are several factors that may lead to the development of VF in HIV/HCV infected patients that are also commonly described in patients with HIV infection alone: the scarce or lack of adherence [29, 30] unknown drug-drug interactions or not declared intake of drugs or substances that may decrease the bio-availability and consequently the efficacy of one or more anti-retrovirals [31] or emergence of drug resistance [32–34].

We cannot exclude that our patients with HIV-VF were less adherent to ART respect to patients with VB or VS, also because they had a clinical history with higher frequency of previous VF respect to the counterpart of VB and VS patients.

However, previous reports [35, 36] showed that HIV medication adherence is improved during DAAs by the greater frequency of visits or contacts with the health care system. We did not administer a specific questionnaire to precisely evaluate the degree of adherence and/or the possible use of illicit substances that could modify the kinetic of the drugs as part of the ART regimen; in this context, physicians posed particular attention to this phenomenon during the clinical follow-up, by asking and reporting in the visit chart any compliance deviation and use of substances. In this regard, two of 13 VF patients admitted a scarce compliance to ART treatment but not to DAAs, while eleven of VF patients were likely adherent to ART treatment.

One other possibility for VF in these patients, is that HIV/HCV infection has a dynamic profile similar to that described in HBV/HCV infection during DAAs treatment.

Of note, HBV rebound might occur following DAA-induced HCV clearance and HBV reactivation was reported in patients with both chronic and resolved HBV infection treated with DAAs [37].

The underline mechanism for this phenomenon has not been precisely established.

Treatment with DAAs, inducing a rapid and profound clearance of HCV, may lead to the loss of viral interference that is mediated by innate or adaptive immune responses [38] with a consequent trigger of HBV replication. Although a direct viral interference between HCV and HIV is controversial, a transient perturbation of immune response consequent to the early decline of HCV load, could be responsible for the HIV-positive viremia in some patients.

In this regard, a recent report evaluating T-cell and monocyte activation over the course of HCV direct-acting antiviral (DAA) therapy in HCV/HIV coinfection [39] showed that after therapy CD4 T-cell activation positively correlated with monocyte activation. This phenomenon did not exist before DAAs therapy and was possibly related to restored liver function or residual immune activation.

Concerning the emergence of HIV drug resistance, as main factor in inducing VF, several recent studies [40–42] have found that low-level viral replication in patients receiving combination ART may promote the selection of drug-resistance mutations, which could negatively impact future ART options.

We showed that, among 7 patients with an available test of resistance at VF, 5 (61%) had RAMs to current ART treatment. Interestingly, PT1 who had a high level score for RAMs to raltegravir, maintained his ART and remained suppressed until the last visit available in May 2019.

Concerning the dynamic of HIV-viremia during DAAs, the only study [19] available in the real world setting of HIV/HCV patients, showed that among 135 patients with HIV-RNA load quantified during DAAs, 15 (11.1%) patients had a transient increase in HIV-RNA, but <100 copies/mL (defined as a blip) and the frequency was similar in the subjects who switched or not ART before DAAs (8% vs. 13% P = 0.45). However, none of these 135 patients had a persistent loss of HIV viral suppression.

In clinical trials on DAAs treatment in HIV/HCV patients [12, 17, 18] no HIV virological failure was reported during anti-HCV therapy, although one or more blips in HIV viremia was detected in these studies during the DAAs treatment.

Prior reports [43, 44] focusing on the archived HIV-DNA, showed that HIV/HCV co-infected patients with successful combination-ART experienced a significant cellular HIV-DNA increase after the start of DAA treatment, suggesting that fast HCV-RNA clearance influence the cellular reservoir and that mobilization of HIV-1 from tissue such as liver and lymph nodes, could be responsible for the increased level of HIV-DNA during DAAs treatment.

Although we did not evaluate the archived HIV-DNA, we had evidence of HIV replication after a dramatic and early decrease of HCV load to undetectable levels.

Possibly, this phenomenon reflects a frailty in regard of HIV viral control with an activation of replicative-competent virus rather than the mobilization from the cellular reservoir. We cannot rule out the possibility of defective viruses-replication; however, the finding of RAMs in some of our patients with VF, suggests a drug-induced selection of genetically competent variants.

We did not specifically evaluate the comedication that is usually related to increasing age and comorbidities; therefore, the association we found between VF and older age in our group of patients, could be the consequence of reduced adherence or drug-drug interactions due to polypharmacy. One other possible explanation for this finding is that older patients could be exposed to suboptimal therapy especially in the pre-HAART era, with accumulation of RAMs.

Altogether, the findings reported in the present study add some interesting information to the issue of virological control of HIV infection during and after current HCV treatment and are consistent with the very scarce literature on this topic.

Our study has some limitations. It has been performed in a single clinical center and has an observational and retrospective nature. The higher incidence rate of VF we detected respect to previous studies could be the consequence of the lower cut-off (50 copies/mL) we used for the definition of virological failure. Additionally, as HIV-1 RNA was much more frequently monitored during DAA treatment than during non-DAA periods, this might also have increased the probability of detecting VF. However, some of our patients with VF had documented RAMs and changed ART regimen; therefore, the closer monitoring of HIV-1 load allowed us to early identify VF.

In conclusion, HIV viremia persisted suppressed in the large majority of HIV/HCV coinfected patients treated with DAAs.

The risk of VF was associated with older age, previous HIV VF or blips and with RV at DAAs initiation, suggesting that a close monitoring of HIV load in selected patients could help to identify a suboptimal antiretroviral therapy. Whether this phenomenon is triggered by the rapid and profound clearance of HCV remains to be established by a mechanistic approach.

## Supporting information

S1 Data (CSV)

S2 Data (CSV)
